# Honesty and dishonesty in gossip strategies: a fitness interdependence analysis

**DOI:** 10.1098/rstb.2020.0300

**Published:** 2021-11-22

**Authors:** Junhui Wu, Szabolcs Számadó, Pat Barclay, Bianca Beersma, Terence D. Dores Cruz, Sergio Lo Iacono, Annika S. Nieper, Kim Peters, Wojtek Przepiorka, Leo Tiokhin, Paul A. M. Van Lange

**Affiliations:** ^1^ CAS Key Laboratory of Behavioral Science, Institute of Psychology, Chinese Academy of Sciences, Beijing 100101, People's Republic of China; ^2^ Department of Psychology, University of Chinese Academy of Sciences, Beijing 100049, People's Republic of China; ^3^ Department of Sociology and Communication, Budapest University of Technology and Economics, H-1111 Budapest, Hungary; ^4^ CSS-RECENS, Centre for Social Sciences, H-1097 Budapest, Hungary; ^5^ Department of Psychology, University of Guelph, Guelph, Ontario, N1G 2W1, Canada; ^6^ Department of Organization Sciences, Vrije Universiteit Amsterdam, 1081HV Amsterdam, The Netherlands; ^7^ Department of Sociology/ICS, Utrecht University, 3584CS Utrecht, The Netherlands; ^8^ University of Exeter Business School, Exeter EX4 4PU, UK; ^9^ Human Technology Interaction Group, Eindhoven University of Technology, 5600MB Eindhoven, The Netherlands; ^10^ Department of Experimental and Applied Psychology, Vrije Universiteit Amsterdam, 1081BT Amsterdam, The Netherlands

**Keywords:** reputation, cooperation, dishonest gossip, fitness interdependence, modelling approach

## Abstract

Gossip, or sharing information about absent others, has been identified as an effective solution to free rider problems in situations with conflicting interests. Yet, the information transmitted via gossip can be biased, because gossipers may send dishonest information about others for personal gains. Such dishonest gossip makes reputation-based cooperation more difficult to evolve. But when are people likely to share honest or dishonest gossip? We build formal models to provide the theoretical foundation for individuals' gossip strategies, taking into account the gossiper's fitness interdependence with the receiver and the target. Our models across four different games suggest a very simple rule: when there is a perfect match (mismatch) between fitness interdependence and the effect of honest gossip, the gossiper should always be honest (dishonest); however, in the case of a partial match, the gossiper should make a choice based on their fitness interdependence with the receiver and the target and the marginal cost/benefit in terms of pay-off differences caused by possible choices of the receiver and the target in the game. Moreover, gossipers can use this simple rule to make optimal decisions even under noise. We discuss empirical examples that support the predictions of our model and potential extensions.

This article is part of the theme issue ‘The language of cooperation: reputation and honest signalling’.

## 1. Introduction

Humans recurrently encounter situations characterized by conflicting interests with ingroup members, outgroup members or strangers. In such mixed-motive situations, the decision to cooperate or compete has important consequences for the involved parties [1,2]. Promoting cooperation in such situations is especially important, because of the benefits of cooperation for collective welfare. Yet, encouraging every individual to cooperate by paying personal cost to benefit others is often challenging [3]. This paradox has stimulated researchers from various disciplines to investigate pathways that facilitate cooperation. One factor that has captured researchers' attention is gossip, which refers to the process of sharing information that is positive, negative or neutral about absent others with one or more receivers [4–9]. Researchers increasingly realize the important social functions of gossip [9–14]. Specifically, to the extent that gossip provides information about others’ trustworthiness, it allows receivers of gossip to detect potential cheaters and selectively cooperate with deserving others [5,15–17]. Moreover, when people are unable to directly punish free riders, gossip can be used as a low-cost form of punishment that can impose reputational costs on free riders [15,18]. Of course, the fact that gossip involves low cost does not mean that it is risk-free: when the gossip target finds out the identity of the gossiper who shares the negative information, they may punish or ostracize the gossiper [19]. In sum, there is a growing body of research suggesting that gossip is a key mechanism that promotes and sustains cooperation [14,20–27].

However, there is at least one important barrier to gossip serving its functions in everyday life: people may be motivated to share dishonest information about others for personal benefit. The possibility that gossip can be biased or dishonest [28,29] may make it difficult for reputation-based cooperation to evolve [9,30,31]. For example, when people manipulate gossip in ways that result in free riders having a good reputation, free riders may use this good reputation to mislead and exploit cooperators. Similarly, if cooperators are falsely assigned bad reputations, an otherwise potentially cooperative interaction between two cooperators may break down because they do not trust each other, leading to mutual defection and confirming the (initial false) bad reputations [32]. Indeed, research shows that gossip does not stimulate cooperation if it is false or inaccurate at sufficiently high levels [11,33,34], notwithstanding the evidence that in certain situations, inaccurate gossip can still support cooperation [35,36]. Because the positive impact of gossip on cooperation thus seems limited to situations in which gossip reflects true information about others, it is important to shed light on the question of when gossip is likely to be honest or dishonest.

Here, we use a novel approach to model and analyse honest and dishonest gossip as a strategic behaviour in line with models from biological signalling theory [37,38]. The key insight of signalling theory is that signals are adaptations shaped by marginal costs and marginal benefits of different behaviours, and that the ultimate function of the signaller's behaviour is to maximize their fitness [39,40]. The goal of ‘honest signalling’ models is to analyse the conditions under which this fitness optimization will result in an honest equilibrium [38,41–43], such that the receiver receives reliable information about the signaller from the signals, and this information in turn helps the receiver to achieve higher fitness. While seminal signalling models investigate pairwise interactions between signallers and receivers [37,38,41], the bare minimum for gossip is a triad of a signaller (i.e. gossiper), a receiver and a target. This implies that while our basic approach is the same, conclusions from seminal signalling models cannot be directly transferred to gossip.

In addition, our approach differs from the traditional approach of indirect reciprocity models that address the effect of noise on the evolution of cooperation [30,35,44]. Noise is usually modelled in terms of errors (e.g. an error of perception or error of judgement in assigning reputations) in these models. The conclusion of these models is that a reputation system that supports the evolution of cooperation is usually robust up to a (not too high) level of noise [30,35,44]. The key difference between the strategic approach and the ‘dishonesty as noise’ approach is that the level of noise is due to external factors and it is usually assumed to be fixed [30,35,44]. By contrast, the frequency of a strategy is determined by internal factors, and a successful strategy will spread through the population. For instance, a dishonest gossip strategy can increase its frequency in the population if it is more successful than an honest one. In the long term, this can challenge the evolution of cooperation even if the frequency of dishonest gossip strategy is low at the start of a simulation [34].

All in all, we propose that it is important to analyse honest and dishonest gossip as a strategic behaviour shaped by marginal costs and marginal benefits of different behaviours. In order to describe these marginal costs and marginal benefits in a gossip triad, we draw on recent theoretical developments on fitness interdependence [45–48]. Specifically, we propose that gossipers will choose a gossip strategy (i.e. honest or dishonest gossip) that maximizes their fitness benefits and minimizes their fitness costs, which are in part determined by the levels of fitness interdependence between the gossiper and the other two parties (i.e. the target and the receiver) that together constitute the gossip triad. Different from a recent review on how interdependence among the actors in the gossip triad affects when people do not gossip [49], we analyse the conditions under which people share honest versus dishonest gossip—a topic that has hardly received any attention—and thus provide a novel and meaningful contribution to the theoretical developments on gossip honesty. In the following sections, we first outline the fitness interdependence perspective and illustrate the types of cues that people use to infer their fitness interdependence with others. We then specify how fitness interdependence between the gossiper and the other two parties in the gossip triad may relate to the gossiper's honest or dishonest gossip strategy using a modelling approach. We end by discussing empirical examples that support the predictions of our models, and potential extensions in future research.

## 2. Fitness interdependence and the situational cues for fitness interdependence inferences

Fitness interdependence refers to the extent to which one or several organisms influence each other's success in replicating their genes [45–48]. Fitness interdependence is slightly different from interdependence theory [50,51] and functional interdependence theory [52]: the latter two theories categorize social situations into several dimensions of how people affect one another (e.g. the degree to which each person can determine their own outcomes; covariation of interests), whereas fitness interdependence focuses solely on fitness interests. While (functional) interdependence theories primarily focus on the proximate mechanisms of behaviours, the concept of fitness interdependence extends these theories by focusing on the ultimate causes of behaviours [53]. Fitness interdependence can be reflected in a stake index (*s*), which depicts the extent to which changes in one's fitness relate to changes in another's fitness [48]. For instance, imagine two people who must work together for their livelihood—each one's well-being depends on the other doing well. More formally, imagine someone paying a personal cost *c* (*c* > 0) to provide a benefit *b* to a recipient, but at the same time also gaining a secondary benefit (*sb*) that is a function of the recipient's gain. When *sb* > *c*, a larger value of *s* suggests that the individual will be more likely to act altruistically, i.e. to provide a benefit to the recipient despite the personal cost this entails. Similarly, welfare tradeoff ratio (WTR), which refers to the extent to which a person values another's welfare relative to their own [54], can be considered a proximal mechanism driven by fitness interdependence. Thus, the higher fitness interdependence one has with someone else, the higher one's WTR towards this other person. Accordingly, the condition for altruistic behaviour involving WTR (i.e. WTR × *b* > *c*) is the same as that involving the stake (i.e. *sb* > *c*). Notably, *s* has a range of values that have different meanings. Positive fitness interdependence (*s* > 0) implies that individuals positively affect one another's survival and reproduction, whereas negative fitness interdependence (*s* < 0) implies that individuals negatively affect one another's survival and reproduction. Of course, there can also be an absence of fitness interdependence (*s* = 0) such that individuals do not have any effect on one another's survival and reproduction.

Notably, it is often challenging to measure how individuals' behaviour affects their own and others’ fitness. If this is the case, how do people assess their fitness interdependence with others? Researchers suggest that humans may have evolved to use various situational cues to assess how their fate is evolutionarily intertwined with others [45]. First, group membership may be one such cue for people to assess their fitness interdependence with others. According to the bounded generalized reciprocity perspective [55,56], human groups provide a container for generalized exchange network, in which individuals who behave cooperatively towards ingroup members gain a good reputation, and thus obtain indirect benefits from other group members. Thus, groups are important for individuals' survival and reproductive success. Indeed, numerous studies have documented that group membership plays an important role in fostering individuals’ trust, cooperation, and norm enforcement behaviours. For example, people are more likely to trust ingroup members [16], cooperate with ingroup members [16,57], and punish in ways that protect the ingroup victims from norm violations, as well as more harshly punishing norm violators who are members of an outgroup than the ingroup [58–61]. These cooperative interactions create fitness interdependence among each other, in that people have an interest in their group's persistence so that these interactions can continue [62]. Thus, it is plausible that compared with an outgroup member, people may be more likely to treat an ingroup member as someone they have positive fitness interdependence with.

Second, individuals’ observable actions towards others in social interactions may also signal their fitness interdependence with others. For instance, when an individual incurs a cost to help another, this helping behaviour can signal that the helper values the recipient, and has enough stake in the welfare of the recipient who may repay with subsequent trust, implying that the helper has positive fitness interdependence with the recipient [54,63]. Third, partners' emotion expressions can be used to assess one's fitness interdependence with partners. For instance, partners' anger expressions may reflect negative fitness interdependence [64,65], whereas partners’ emotions of happiness, forgiveness or gratitude may reflect positive fitness interdependence and a higher likelihood of helping [66–68].

## 3. Fitness interdependence and gossip strategies: a modelling approach

**Figure 1.**
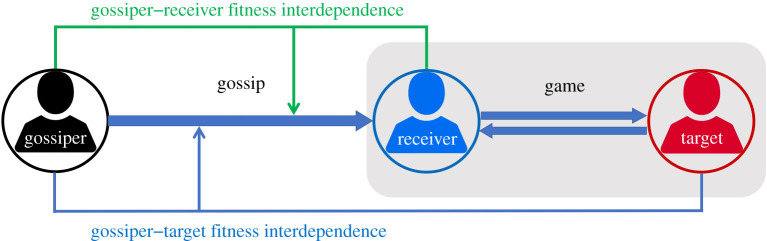
Structure of the sequential interaction between the gossiper, the receiver and the target. First, the gossiper decides to send honest or dishonest gossip to the receiver, then the receiver and the target interact in a two-person game. The gossiper's fitness outcome (and thus their optimal decision about whether to share honest gossip) is influenced by their fitness interdependence with the receiver and the target.

Drawing on the fitness interdependence framework, we model the gossiper's gossip behaviour towards the receiver in the gossip triad based on the gossiper's fitness interdependence with the target and the receiver (figure 1). We assume that the receiver and the target play a one-shot two-person game. The receiver does not know the target's behavioural type until the gossiper sends them this information. It is in the interest of the receiver to find out the target's type, as the receiver's optimal response may depend on this information. In our theoretical models, we make the following simplifying assumptions: (i) the target is either a cooperating type (always cooperate) or a defecting type (always defect); (ii) the gossiper has accurate knowledge about the target's type that is acquired through experience or direct observation, and always sends gossip that either honestly informs or misleads the receiver about the target's type; (iii) the receiver will trust any gossip that they receive, and (iv) there is no noise (i.e. unintended errors that cause discrepancies between the expected outcomes and actual outcomes; [69]) in the gossip transmission process. This allows us to investigate the conditions under which the gossiper transmits honest or dishonest gossip about the target's type. Later on, we relax the assumption of noise. Modification to the other assumptions is an area that can be addressed in future extensions of our work, but is beyond the scope of the current paper.

Below, we analyse four possible games that are played between the receiver and the target: a stag-hunt game, a snowdrift game, a helping game and a punishment game (see electronic supplementary material for a detailed description of the games). These four games provide examples for four types of possible outcomes of honest gossip for the fitness of the receiver and the target: (i) mutually beneficial (e.g. stag-hunt game with a cooperating target) [70], (ii) beneficial for the receiver but costly for the target (e.g. snowdrift game with a cooperating target) [71], (iii) beneficial for the target but costly for the receiver (e.g. helping game with a cooperating target) [72], and finally (iv) mutually costly (e.g. punishment game with a defecting target) [73].

### (a) The model

The receiver (r) and the target (t) play a two-person game *F* = (*B*, *E*), where *E* denotes the payoffs from the game and *B* denotes the strategy set available to the players. The gossiper (g) is not involved in this game, but has a stake in the receiver's and the target's fitness outcomes, which corresponds to the gossiper's fitness interdependence with the receiver and the target. Figure 1 shows the structure of the sequential interaction between the gossiper, the receiver and the target.

Let *V*_gr_ and *V*_gt_ denote the fitness interdependence of the gossiper with the receiver and the target, respectively. Accordingly, the gossiper's fitness outcome as a result of the interaction between the receiver and the target can be written as follows:

3.1 $$E_{g}=V_{\mathrm{gr}}E_{r}+V_{\mathrm{gt}}E_{t}.$$

Here, *E_*g*_*, *E_*r*_* and *E_*t*_* denote the fitness outcomes for the gossiper, the receiver and the target, respectively, for which we use payoffs in the game as proxy measures. The gossiper's action of sharing honest (H) or dishonest (D) gossip will influence the receiver's behavioural strategy towards the target (*B*_r_), which not only influences the target and receiver's fitness, but also influences the gossiper's fitness via the varying levels of fitness interdependence. Sharing honest gossip will be an equilibrium strategy if the fitness outcome of gossipers sharing honest gossip is higher than the fitness outcome of gossipers sharing dishonest gossip, i.e. *E*_g_(H) > *E*_g_(D).^1^ Accordingly, the following condition must hold in the honest gossip equilibrium:

3.2 $$V_{\mathrm{gr}}E_{r}(H)+V_{\mathrm{gt}}E_{t}(H)>V_{\mathrm{gr}}E_{r}(D)+V_{\mathrm{gt}}E_{t}(D).$$

Here, *V*_gr_*E*_r_(H) or *V*_gr_*E*_r_(D) describes the influence of the receiver's fitness outcome on the fitness outcome of the gossiper via the fitness interdependence between the gossiper and the receiver, assuming gossip is honest or dishonest; *V*_gt_*E*_t_(H) or *V*_gt_*E*_t_(D) describes the influence of the target's fitness outcome on the fitness outcome of the gossiper via the fitness interdependence between the gossiper and the target, assuming gossip is honest or dishonest. We investigate the conditions for honest gossip (based on inequality (3.2)) across four different two-person games (i.e. stag-hunt game, snowdrift game, helping game and punishment game; see electronic supplementary material, figure S1). Table 1 provides an overview of the conditions for honest gossip in these four games with cooperating and defecting targets (details of deriving these conditions can be found in the electronic supplementary material).

**Table 1.** Conditions for honest gossip across four games with a cooperating or defecting target. In the stag-hunt and snowdrift games, *R* denotes the benefit of mutual cooperation, *P* denotes the cost of mutual defection, *T* is the ‘temptation’ benefit of defecting against a cooperating player, while *S* is the ‘sucker's pay-off’; in the helping and punishment games, *c* denotes the cost of helping/punishing the target, *b* denotes the benefit for the target from helping and *γ* denotes the fine imposed on the target. *V*_gr_, the gossiper's fitness interdependence with the receiver; *V*_gt_, the gossiper's fitness interdependence with the target.

game	target type	condition for honest gossip	
stag-hunt game	cooperating	$V_{\mathrm{gr}}(R-T)>V_{\mathrm{gt}}(S-R)$	inequality (3.3)
	defecting	$V_{\mathrm{gr}}(P-S)>V_{\mathrm{gt}}(T-P)$	inequality (3.4)
snowdrift game	cooperating	$V_{\mathrm{gr}}(T-R)>V_{\mathrm{gt}}(R-S)$	inequality (3.5)
	defecting	$V_{\mathrm{gr}}(S-P)>V_{\mathrm{gt}}(P-T)$	inequality (3.6)
helping game	cooperating	$V_{\mathrm{gt}}b>V_{\mathrm{gr}}c$	inequality (3.7)
	defecting	$V_{\mathrm{gr}}c>V_{\mathrm{gt}}b$	inequality (3.8)
punishment game	cooperating	$V_{\mathrm{gr}}c>-V_{\mathrm{gt}}\gamma$	inequality (3.9)
	defecting	$-V_{\mathrm{gr}}c>V_{\mathrm{gt}}\gamma$	inequality (3.10)

**Figure 2.**
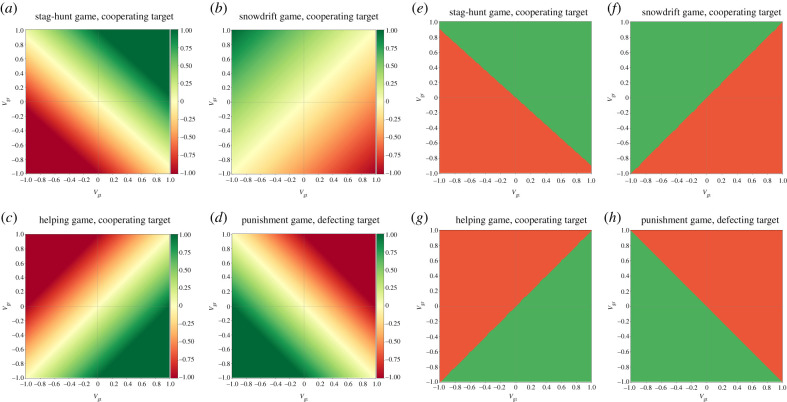
The marginal cost/benefit of honest gossip (*a–d*; darker green: higher marginal benefit of honest gossip, darker red: higher marginal cost of honest gossip; cost and benefit are represented with negative and positive numbers on the right bar) and the predicted behaviour of the gossiper (*e–h*; green area: honest gossip, red area: dishonest gossip) across the four main interaction types as a function of the fitness interdependence between the gossiper and the target (*V*_gt_) and between the gossiper and the receiver (*V*_gr_). (*a,e*) Mutualism (*receiver*/*target*: +/+; stag-hunt game with a cooperating target; *T* = 0, *S* = 0.1); (*b,f*) antagonism (*receiver*/*target*: +/−, snowdrift game with a cooperating target; *T* = 1.5, *S* = 0.5); (*c,g*) antagonism (*receiver*/*target*: −/+, helping game with a cooperating target; *b* = 1, *c* = 1); (*d,h*) competition (*receiver*/*target*: −/−, punishment game with a defecting target; *c* = 1, *γ* = 1).

We have investigated four types of interactions in two-person games between the receiver and the target in the gossip triad. Our models show that the gossiper's fitness interdependence with the target and the receiver differentially affects the gossiper's likelihood to send honest or dishonest gossip across different types of interactions between the receiver and the target: (i) when mutual cooperation is beneficial for both the receiver and a cooperating target (i.e. the stag-hunt game versus a cooperating target), the gossiper will be honest when their overall fitness interdependence with the receiver and the target is positive (figure 2*a,e*); (ii) when defecting with a cooperating target is beneficial for the receiver, but costly for the target (i.e. the snowdrift game versus a cooperating target), the gossiper will be honest when their fitness interdependence with the receiver is higher than that with the target (figure 2*b,f*); (iii) when helping a cooperating target is costly for the receiver but beneficial for the target (e.g. the helping game versus a cooperating target), the gossiper will be honest when their fitness interdependence with the target is higher than that with the receiver (figure 2*c,g*); (iv) when punishing a defecting target decreases the pay-off of both the receiver and the target (e.g. the punishment game versus a defecting target), the gossiper will be honest when their overall fitness interdependence with the receiver and the target is negative (figure 2*d,h*). The slope of the boundary between the honest and dishonest gossip regions is determined by the marginal cost/benefit of honest gossip for the receiver and the target, respectively (e.g. the values of *c* and *b* in the helping game). The slope will be at the main diagonal when the marginal cost and benefit have the same value (e.g. *c* = *b* = 1). When there is no marginal benefit or cost for the receiver or the target in choosing one action over another action, then the gossiper's fitness interdependence with the receiver or the target does not matter. For example, if there is no marginal cost of being suckered in the snowdrift game (for the target, i.e. *R* = *S*), then the gossiper's fitness interdependence with the target does not matter (i.e. horizontal slope). If helping is cost-free in the helping game (*c* = 0), then the gossiper's fitness interdependence with the receiver does not matter (i.e. vertical slope, see examples for variations in marginal cost/benefit in the electronic supplementary material, figures S2–S9).

Taken together, our models across four different games illustrate that the gossiper's action will be determined by two broad factors: (i) the gossiper's fitness interdependence with the receiver and the target, and (ii) the marginal cost/benefit (pay-off differences between the two possible options) for the receiver and the target (i.e. game type). This implies that in order to predict the gossiper's behaviour, it is essential to have information about both factors.

However, upon closer investigation (see details in the electronic supplementary material), our models suggest that there is a simple rule that allows the gossiper to make optimal decisions in certain situations without being able to judge the exact parameters of the game between the receiver and the target. Table 2 shows honest and dishonest gossip based on different games and the valence of the gossiper's fitness interdependence with the receiver and the target. The rule is very simple: when there is a perfect match between fitness interdependence and the effect of honest gossip (i.e. *V*_gr_ and *E*_r_(H) are both positive or negative, and *V*_gt_ and *E*_t_(H) are both positive or negative), the gossiper should always be honest; when there is a perfect mismatch (i.e. *V*_gr_ and *E*_r_(H) are opposite in valence, and *V*_gt_ and *E*_t_(H) are opposite in valence), then the gossiper should always be dishonest. When there is a partial match between fitness interdependence and the effect of honest gossip, the gossiper has to make a choice based on the marginal cost/benefit and their fitness interdependence with the receiver and the target; these are the situations in which the knowledge (estimation) of marginal cost/benefit is important (table 2). Notably, people can assess fitness interdependence and marginal cost/benefit using various cues (e.g. expression of emotions [45,52,78]), so that while they might not know the exact levels of fitness interdependence or marginal cost/benefit, they probably have some reasonable approximation thereof.

**Table 2.** Summary of predictions of the gossiper's behaviour depending on the game type and fitness interdependence. *V*_gr_, the gossiper's fitness interdependence with the receiver; *V*_gt_, the gossiper's fitness interdependence with the target; *E*_r_(H), the fitness outcome of honest gossip for the receiver; *E*_t_(H), the fitness outcome of honest gossip for the target. +/–, positive/negative.

game type	fitness interdependence		effect of honest gossip		gossiper's expected behaviour	
	*V*_gr_	*V*_gt_	*E*_r_(H)	*E*_t_(H)		
stag-hunt game^a^	+	+	+	+	honest	
	+	–	+	+	honest if inequality (3.3) holds	dishonest otherwise
	–	+	+	+	honest if inequality (3.3) holds	dishonest otherwise
	–	–	+	+	dishonest	
snowdrift game^a^	+	+	+	–	honest if inequality (3.5) holds	dishonest otherwise
	+	–	+	–	honest	
	–	+	+	–	dishonest	
	–	–	+	–	honest if inequality (3.5) holds	dishonest otherwise
helping game^a^	+	+	–	+	honest if inequality (3.7) holds	dishonest otherwise
	+	–	–	+	dishonest	
	–	+	–	+	honest	
	–	–	–	+	honest if inequality (3.7) holds	dishonest otherwise
punishment game^b^	+	+	–	–	dishonest	
	+	–	–	–	honest if inequality (3.10) holds	dishonest otherwise
	–	+	–	–	honest if inequality (3.10) holds	dishonest otherwise
	–	–	–	–	honest	

^a^Versus a cooperating target.

^b^Versus a defecting target.

**Figure 3.**
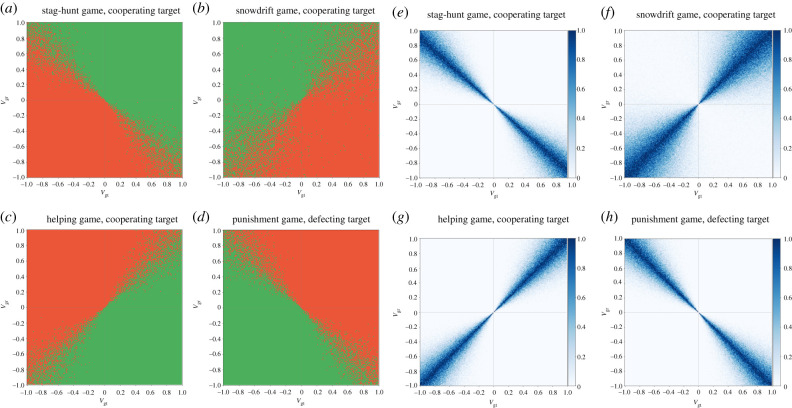
The predicted behaviour of the gossiper under noise (*a–d*; green area: honest gossip, red area: dishonest gossip) and the probability of making a mistake (*e–h*; *µ* = 0; σ = 0.25; see further details in the electronic supplementary material). (*a,e*) Mutualism (*receiver*/*target*: +/+; stag-hunt game with a cooperating target; *T* = 0, *S* = 0.1); (*b,f*) antagonism (*receiver*/*target*: +/−, snowdrift game with a cooperating target; *T* = 1.5, *S* = 0.5); (*c,g*) antagonism (*receiver*/*target*: −/+, helping game with a cooperating target; *b* = 1; *c* = 1); (*d,h*) competition (*receiver*/*target*: −/−, punishment game with a defecting target; *c* = 1, *γ* = 1).

What happens when the estimation of the marginal cost/benefit (e.g. game parameters) is not perfect? In order to answer this question, we add noise to the estimation of the relevant parameters (*S*, *T*, *c*, *b* and *γ*) in the models. We assume that noise is drawn from a normal distribution (*f*) with a mean *µ* and standard deviation σ (see details in the electronic supplementary material, figures S10–S18). Figure 3 shows the effect of noise on the decisions made by the gossiper (figure 3*a–d*) and the probability of making a mistake (i.e. being dishonest when the optimal decision is to be honest and *vice versa*; figure 3*e–h*). Noise creates a fuzzy edge between areas for honest and dishonest gossip, and the probability of making a mistake is the highest near the edge of these areas. Moving further away from the edge, this probability drops to zero. How fast this probability drops to zero depends on two factors (see further details in the electronic supplementary material): (i) the magnitude of noise, i.e. the higher the σ, the larger is the area affected by noise; (ii) the incentive structure of the game, i.e. the higher the marginal benefit to be honest (or the marginal cost to be dishonest), the less important is noise (see examples in the electronic supplementary material). Note that the quadrants that include the edges are ones with partial match between fitness interdependence and marginal cost/benefit, and the effect of noise is the most prominent in these quadrants (given reasonable values). This effect is in line with our previous conclusion: gossipers can make optimal decisions in the quadrants of perfect match or perfect mismatch even if they do not know the exact values of game parameters. Similar conclusions can be drawn when there is noise in estimating fitness interdependence (see electronic supplementary material, figure S19) or in estimating both game parameters and fitness interdependence (see electronic supplementary material, figure S20).

### (b) Overview of related literature and implications for future research

To our knowledge, there are no extant models that have tested the optimal gossip strategies in situations varying in fitness interdependence. Our models provide a functional explanation for why people tend to share honest or dishonest information, which we believe will benefit future empirical research on the proximate causes of honest and dishonest gossip behaviour. Although we are not aware of any study that has directly tested the effect of fitness interdependence on honest or dishonest gossip behaviour, some studies do suggest negative or positive fitness interdependence between the gossiper and the target. For instance, gossiping about rivals is indicative of negative fitness interdependence, whereas gossiping about friends or loved ones is a strong indication of positive fitness interdependence [36]. Strong negative emotions (e.g. anger) also suggest negative fitness interdependence [64,65]. The few studies that could be evaluated based on our criteria show a pattern consistent with our predictions (see electronic supplementary material, table S1).

Negative fitness interdependence between the gossiper and the target (e.g. the gossiper and the target being rivals or competitors) makes the gossiper more likely to dishonestly describe a target's action as bad when it is good (e.g. describing a cooperative person as a free rider), and to honestly describe the target's action as bad when it is indeed bad (e.g. describing a free rider as they are) [79,80]. Similar observations were made when there was potential negative interdependence between the gossiper and the receiver (e.g. they were rivals): the gossiper was more likely to be dishonest if the effect of honest gossip was positive for the receiver [81]. By contrast, positive honest gossip (gossip that benefits the target) is more likely to be shared about targets with whom the gossiper has positive fitness interdependence (e.g. friends or lovers, [79]). In line with our predictions, negative emotions indicative of potential negative fitness interdependence are more likely to lead to honest gossip when honest gossip negatively affects the target [22].

Another potential indicator of fitness interdependence is group membership. A recent study [36] found that gossipers were more likely to share dishonest gossip with outgroup receivers (i.e. receivers with potentially negative fitness interdependence) when honest gossip positively affected the receivers (i.e. it would have increased the receivers' pay-off, see [36]). Conversely, gossipers were more likely to share honest gossip with ingroup receivers when honest gossip had positive consequences for the receivers [36].

All in all, our modelling results suggest that the optimal gossip strategy varies according to the marginal costs and benefits resulting from honest gossip and the gossiper’s fitness interdependence with the other actors in the gossip triad. A case in point is that people adopt a dishonest strategy to share only cooperative reputation information about related others (e.g. children) to protect their outcomes, while they adopt honest strategies about unrelated others (e.g. sharing non-cooperative information).

One novelty of our model is that it can also be used as a baseline against which to evaluate broader proximate mechanisms, often taking the forms of general principles of human behaviour. Notably, many societies have developed strong norms, sometimes even formal rules, for how to deal with (non-public) information, such that honest strategies may sometimes be adopted regardless of the optimal strategies in a certain situation. An example is the norm or cultural wisdom ‘honesty is the best policy’ (Benjamin Franklin). We recommend and envision research programmes that provide critical tests of normative influences on human behaviour, which might (or might not) overrule the pressure of fitness interdependence. Future research can use insights from our models to test predictions about when gossip is likely to be honest or dishonest. While it may not be possible to estimate the exact values of fitness interdependence, proxies such as rivalry, competition, friendship, group membership, negative or positive emotions could be used to predict the valence (negative versus positive) of fitness interdependence [45].

## 4. Conclusion

A growing body of research has begun to highlight the importance of gossip honesty in promoting and sustaining cooperation. Yet, the field is still in the early stages of understanding the situational underpinnings of individuals' strategies to share honest or dishonest gossip. Here, we draw on recent theoretical developments on fitness interdependence to build formal models to predict when people are likely to share honest or dishonest gossip about a target's behavioural type (i.e. cooperating versus defecting type) depending on the levels of fitness interdependence between the gossiper and the other two parties in the gossip triad, as well as the type of games that the receiver is about to play with the target. We show that honesty is determined by the marginal cost/benefit resulting from honest or dishonest gossip. These findings are consistent with the results of earlier work on signalling games [41,82]. We also show that there is a simple ‘matching rule’ between the valence of fitness interdependence and the effect of honest gossip: gossipers should always be honest when there is a perfect match and they should be dishonest when there is a perfect mismatch. This simple rule allows gossipers to make an optimal decision even if there is noise in estimating fitness interdependence and the effect of honest gossip. Our models are guided by the scientific principle of parsimony, but we believe that future research can extend our models by relaxing some assumptions and include complexity that is part of interdependence in everyday life. In addition to noise, social network structures may also be important in guiding individuals' gossip behaviour. Further research can address these questions by exploring how cues of fitness interdependence between the gossiper and the target and between the gossiper and the receiver might independently or jointly affect the gossiper's strategies across various interaction contexts using laboratory experiments and scenario studies. More importantly, future theoretical work can extend our models by taking into account other factors, such as social network structures [83].

## Endnote

1 Although there seems to be no difference between our approach (fitness maximization) and classic notions of rationality (e.g. the expected utility theory), these two approaches are different. Expected utility theory prescribes a rational choice that maximizes the expected utility. Yet, humans often encounter environmental, cognitive and biological constraints that restrict information processing [74]. Despite these constraints, humans have been successful in decision-making, because the human mind is equipped with a collection of specialized information processing mechanisms shaped through natural selection to encourage behaviours that have adaptively maximized fitness in the ancestral environment [75,76]. More importantly, the design of these mechanisms is ecologically rational rather than classically rational [77]. For example, humans may have evolved specialized psychological mechanisms to infer their fitness interdependence with others from other situational cues (rivalry, group membership, etc.) [45,52].

## Data accessibility

This article has no additional data.
